# Supplementary material for: Polyketide synthase gene clusters in the ericoid mycorrhizal fungus Oidiodendron maius: genome-wide prediction, expression profiling, and functional characterization of knockout mutants
Source: Front Plant Sci. 2026 Sep 16;17:1924430. doi: 10.3389/fpls.2026.1924430 (PMC13623903; doi:10.3389/fpls.2026.1924430)
Supplement: Supplementary file 1 [file SupplementaryFile1.zip › Supplementary Materials/Supplementary Folder 1.zip!/Oidma1_AssemblyScaffolds_Repeatmasked/knownclusterblast/cluster44/jgi.p_Oidma1_135410_mibig_hits.html]

| MIBiG Protein | Description | MIBiG Cluster | MiBiG Product | % ID | % Coverage | BLAST Score | E-value |
| --- | --- | --- | --- | --- | --- | --- | --- |
| AIG62138.1 | carboxylesterase\_family\_protein | BGC0000120 | Patulin\_biosynthetic\_gene\_cluster | 52 | 100.0 | 77 | 6.2e-15 |
| AAP03116.1 | putative\_carboxyesterase | BGC0000874 | Blasticidin\_biosynthetic\_gene\_cluster | 39 | 98.4848484848 | 54 | 5.6e-08 |
